# Prevalence and factors associated with occupational burnout among HIV/AIDS healthcare workers in China: a cross-sectional study

**DOI:** 10.1186/s12889-016-2890-7

**Published:** 2016-04-14

**Authors:** Zhengxue Qiao, Lu Chen, Mingqi Chen, Xin Guan, Lin Wang, Yang Jiao, Jiarun Yang, Qinghua Tang, Xiuxian Yang, Xiaohui Qiu, Dong Han, Jingsong Ma, Yanjie Yang, Xiuwei Zhai

**Affiliations:** Department of Medical Psychology, Public Health Institute of Harbin Medical University, Harbin, China; Department of Endocrinology, Peking Union Medical College Hospital, Beijing, China; Department of infection control, the First Affiliated Hospital of Harbin Medical University, Harbin, China; Department of Immunization Programs, Heilongjiang Province Center for Disease Control and Prevention, Harbin, China; Guangxi University of Chinese Medicine, Nanning, China; Daqing LongNan Hospital, Daqing, China

**Keywords:** China, Burnout, HIV/AIDS healthcare workers, Psychology

## Abstract

**Background:**

Burnout is a psychosomatic syndrome characterized by three dimensions (emotional exhaustion [EE], feelings of depersonalization [DP], and reduced personal accomplishment [PA]). We determined the prevalence of burnout and mental health status between HIV/AIDS healthcare workers and other healthcare workers, and determined the factors associated with burnout of HIV/AIDS healthcare workers.

**Methods:**

All participants were asked to complete a self-administered questionnaire. The participants were recruited from the departments of infectious diseases in four hospitals which treated HIV/AIDS. The questionnaire included demographics, the Maslach Burnout Inventory-General Survey (MBI-GS), the Symptom Checklist 90 (SCL-90), the Eysenck Personality Questionnaire (EPQ), and the Trait Coping Style Questionnaire (TCSQ).

**Results:**

A total of 512 questionnaires were distributed; 501 questionnaires were completed and collected (the response rate was 97.9 %). After eliminating nine invalid questionnaires (1.80 %), 264 physicians and nurses caring for HIV/AIDS and 228 physicians and nurses caring for other infectious diseases provided valid responses (98.2 %). The HIV/AIDS healthcare workers’ scores on the emotional exhaustion (*F* = 6.350, *p* = 0.012) and depersonalization dimensions (*F* = 8.533, *p* = 0.004) were significantly higher than other healthcare workers. The HIV/AIDS healthcare workers had higher total scores and positive items on the Symptom Checklist 90 (SCL-90) compared with other healthcare workers. Low job satisfaction, serious somatization, interpersonal sensitivity, poor quality of sleep, high psychoticism scores, and use of negative coping styles were frequently associated with burnout.

**Conclusions:**

Burnout was shown to be highly prevalent in HIV/AIDS healthcare workers, 76.9 % of whom met the accepted criteria for burnout. In addition, compared with other healthcare workers, HIV/AIDS healthcare workers experienced lower levels of psychological health. Interventions should be targeted at reducing the occurrence of burnout and alleviating psychological pressure amongst HIV/AIDS healthcare workers.

## Background

Burnout is a psychosomatic syndrome characterized by three dimensions (emotional exhaustion [EE], feelings of depersonalization [DP], and reduced personal accomplishment [PA]) [1]. The focus of research regarding burnout has involved “human service personnel,” such as the police, teachers, and healthcare workers. Currently, burnout is an important issue in healthcare, particularly in emergency medicine [2], obstetrics and gynecology [3], surgery [4], and infectious diseases [5]. These healthcare specialists are at high risk of burnout because of work overload, high work-related demand, and a complex work environment. Human immunodeficiency virus infection and acquired immune deficiency syndrome (HIV/AIDS) are very important diseases in the departments of infectious diseases because HIV/AIDS is incurable, has a high mortality rate, and can seriously impact human health [6]. In recent years, the HIV epidemic blowout problems have aroused great attention in China, especially amongst young students. By the end of 2014, the government had reported that the number of HIV/AIDS patients was 500,679 in China, which is a 16.7 % increase in 1 year. It is a remarkable fact that 10 regions have reported that there are > 100 students living with HIV and the number is on the rise every year. Thus, HIV/AIDS healthcare workers are experiencing work-related pressure, such as work overload and long work hours, like other healthcare workers. In addition, HIV/AIDS healthcare workers have anxiety over safety practices because of the risk of occupational exposure to HIV [7]. Indeed, HIV/AIDS healthcare workers may be at risk of experiencing burnout.

In contrast with other medical staff, HIV/AIDS healthcare workers have an excessive workload, higher levels of stress, and worse working conditions because of the particularity of HIV, which includes infectivity, stigma, and discrimination [8, 9]. In China, people living with HIV and AIDS patients have increased by nearly five-fold over the past decade, but the growth of HIV/AIDS healthcare workers is less than 1-fold. Thus, the ratio between healthcare workers and patients is imbalanced because of a serious shortage of HIV/AIDS healthcare workers. Most AIDS-designated medical hospitals are far away from the city center and in harsh environments, which leads to less contact with the outside world, lack of recreation, and limited time at home, resulting in fewer opportunities to communicate with family among HIV/AIDS healthcare workers. In addition, protective infectious measures against contracting HIV are not perfect [10], which may lead to psychological pressure. Studies have shown that the working environment is an important factor affecting the mental health of healthcare workers [11]. Moreover, the risk of occupational exposure for healthcare workers engaged in AIDS prevention and control work is 31.8 % in China, but only 0.3–0.5 % in Western countries [12]. Most of the physicians and nurses fear HIV and are anxious of contraction when providing treatment and care services to patients. Research in the past few years has shown that burnout among HIV/AIDS healthcare workers has been reported in Western countries [13, 14]. A study on burnout syndrome in healthcare providers of people living with HIV in Brazil showed that 26.4 % had high EE scores, 17.2 % had elevated DP scores, and 10.5 % had decreased PA scores [13]. Therefore, these factors may make healthcare providers more likely to experience burnout in comparison to workers in other healthcare areas.

Of particular importance are the consequences of burnout among HIV/AIDS healthcare workers, including poorer quality of care, decreased empathy, absenteeism, increased turnover, and decreased job performance [13, 15]. Burnout not only endangers the health and well-being of healthcare workers, but also has an adverse effect on the lives of patients and the physician-patient relationship. Burnout causes a decline in patient trust of healthcare workers and compliance, and detracts from the quality and quantity of medical services, the vocational development of healthcare workers, and quality of life [16]. As a result, burnout among HIV/AIDS healthcare workers diminishes the preventive efforts and control work of HIV/AIDS, of which the epidemic cannot be effectively curbed, even if the epidemic is a huge threat to social stability and national security. Therefore, a study involving the prevalence of burnout and mental health among HIV/AIDS healthcare workers, as well as the factors associated with burnout, is warranted. Additionally, it is significant that some effective measures should be implemented to improve the prevention and treatment of the HIV/AIDS epidemic in China.

Due to the adverse consequences of burnout among HIV/AIDS healthcare workers, determining the related risk factors is extremely important. On the basis of a previous study, burnout among healthcare workers is due to a combination of personal and workplace environment factors [17]. These factors include demographics, personality traits, anxiety, depression, emotional reactions, the aptitude for interpersonal relationships, coping strategies, intensity and high expectations in the workplace, and job satisfaction [15, 18]. A Chinese study showed that coping style has a partial mediating effect on the relationship between the Core Self-Evaluation Scale (CSE) and the burnout syndrome among nurses [19]. A longitudinal study of healthcare workers in Australia revealed that coping style is a major determinant of burnout [20]. In addition, there is a close relationship between burnout and the type of individual personality traits. Rodrigo [21] demonstrated that a personality characteristic that provides resistance to stress can protect participants against burnout. Xiao et al. [22] reported that burnout was negatively correlated with intrinsic and extrinsic job satisfaction in the sampled population.

In the current study, we determined the prevalence of burnout among HIV/AIDS healthcare workers and determined the factors associated with burnout in HIV/AIDS healthcare workers. Our findings provide the theoretical basis for the effective promotion of physical and mental health of HIV/AIDS healthcare workers, for decreasing occupational burnout, and for improving work performance in HIV/AIDS treatment.

## Methods

### Study design and participants

The HIV/AIDS healthcare workers are concentrated in departments of infectious diseases in hospitals where HIV/AIDS is treated. Thus, the healthcare workers selected as the sample in this study were recruited from departments of infectious diseases. This cross-sectional study was conducted in Nanning, which is the capital of Guangxi province, and has one of the highest incidences of HIV/AIDS in China, including physicians and nurses from four hospitals that treat HIV/AIDS. We personally contacted the healthcare workers, invited them to participate, and clearly explained the aims and significance of this study, as well as the method by which to complete the questionnaires. Participation was completely voluntary, without any monetary motivation, and each participant signed a written informed consent. We ensured confidentiality and immediately provided an explanation without inducement for any unclear questionnaire items. Self-administered questionnaires were directly distributed to the participants who were allotted 15 min to complete the questionnaire. The questionnaires were collected immediately after completion. We checked the questionnaires to avoid errors and ensure quality. Five hundred twelve questionnaires were distributed and a total of 501 questionnaires were completed and collected (the response rate was 97.9 %), but there were 9 invalid questionnaires (1.80 %). Of the 264 physicians and nurses caring for HIV/AIDS and the 228 physicians and nurses caring for other infectious diseases in the departments of infectious diseases provided valid responses (98.2 %). This study was approved by the Ethics Committee of Harbin Medical University.

### Measures

#### Demographics

The basic socio-demographic information included age, gender, education, marital status. The education was grouped into three types (high school or below, university, and postgraduate). The marital status was categorized as married or single.

#### Maslach Burnout Inventory

Burnout symptoms in this study were assessed by a Chinese version of the 15-item Maslach Burnout Inventory-General Survey (MBI-GS) [23]. The MBI assesses all three dimensions of burnout (emotional exhaustion [EE], depersonalization [DP], and personal accomplishment [PA]), as rated on a 7-point Likert scale (ranging from 0 = never to 6 = every day). The participants were asked to indicate the frequency with which they had been experiencing certain job-related feelings. The cut-off points of the MBI-GS in the Chinese version were as follows: EE (low, < 11; medium, 11–15; and high >15); DA (low, < 9; medium, 9–12; and high, > 12); PA (low < 19; medium, 19–22; and high >22) [24]. The MBI has been translated into Chinese and showed satisfactory reliability and validity; the internal consistency coefficient of the three dimensions was 0.896, 0.747 and 0.825 [19]. In this study, the Cronbach’s alpha for all 15 items was 0.830, and for EE, DP and PA was 0.881, 0.873, and 0.879, respectively.

#### Symptom Checklist 90 (SCL-90)

The scale developed by Derogatis [25, 26] is a 90-item questionnaire that assesses 10 primary psychological symptoms, as follows: somatization (SOM); obsessive-compulsive (O-C); interpersonal sensitivity (INT); depression (DEP); anxiety (ANX); hostility (HOS); phobic anxiety (PHOB); paranoid ideation (PAR); psychoticism (PSY); and sleep (SLE). The scores of each item range from 1 (none) to 5 (severe). The positive symptom scores were between 2 and 5. Any 1 factor with a score > 2 or > 43 positive items can be considered, which is indicative of psychological problems. The SCL-90 has been translated into Chinese and shown to have satisfactory reliability and validity [27, 28]. The internal reliability of each subscale measured by Cronbach’s alpha was in the 0.78–0.86 range [29]. The scale has been widely used as a clinical assessment in many countries, and particularly studied in the field of mental health [28, 30].

#### Eysenck Personality Questionnaire (EPQ)

This study used the Eysenck Personality Questionnaire to assess the personality traits of participants. The scale includes three 12-item indices (psychoticism [P], extraversion [E], and neuroticism [N]), along with a 12-item lie (L) scale [31, 32]. Each item requires a yes-no response. The high score on the P scale indicates that the personality traits are withdrawn, indifferent to others, and even hostile. A high score on the E scale explains emotional instability, manifested as anxiety, irritability, depression, and complained of a variety of physical discomfort. A high score on N explains that the participants are outgoing, optimistic, and sociable. The L scale assesses the tendency to hide true information. The internal reliability of each subscale measured by Cronbach’s alpha was in the 0.74–0.78 range (except for the P subscale [0.60]) [33].

#### Trait Coping Style Questionnaire

The Trait Coping Style Questionnaire (TCSQ) consists of 20 items, rated on a 5-point Likert scale, ranging from 1 to 5 (1 = certainly, 2 = generally, 3 = uncertain, 4 = generally not, and 5 = certainly not) [34]. The TCSQ evaluates the two independent dimensions of coping (negative coping style [NC] and positive coping style [PC]). The scale is the Chinese adaptation of the original test: the validity and reliability have been validated [35]. The Cronbach’s alpha of the positive and negative coping style scales were 0.790 and 0.776, respectively [36].

#### Job satisfaction

The job satisfaction was measured by asking the question, “Are you satisfied with current job” with the following response choices: “1 = satisfactory; 2 = moderately satisfactory; and 3 = unsatisfactory.”

### Statistical analysis

SPSS (version 18.0) was used for statistical analysis. All tests were two-tailed, with a statistical significance level set at a *p* < 0.05. HIV/AIDS healthcare workers and other healthcare workers were included in the analyses. Demographic data, including age, gender, education, marital status, and job satisfaction were recorded as numbers and percentages. Comparisons of burnout for demographics were tested by t-tests and ANOVA. Multivariate analyses of variance (MANOVA) were performed to compare the three dimensions of burnout and the 10 subscales of SCL-90 variables between two groups. The positive items of SCL-90 variables were compared between groups byχ^2^ tests. We used logistic regression to assess the factors associated with burnout symptoms.

## Results

### Socio-demographic data of participants

After excluding 9 invalid questionnaires, 492 participants completed the questionnaires during the survey. Of the 264 physicians and nurses caring for HIV/AIDS and the 228 physicians and nurses caring for other infectious diseases in the departments of infectious diseases provided valid responses. Of these physicians and nurses, 29.9 % were males and 70.1 % were females, and 68.2 % were married and 31.8 % were single. Of the participants, 21.3 % had attained a postgraduate qualification. The average age of the participants was 33.77 years (SD =8.63), with a range of 19–60 years. There was no significant association between burnout scores on age and gender (*p* > 0.05). The scores of the three dimensions of occupational burnout had no significant difference between physicians and nurses (*p* > 0.05).

### Prevalence of burnout symptoms

The prevalence of low EE, DP, and reduced PA was 41.7, 67, and 54.2 %, respectively. The prevalence of medium EE, DP, and reduced PA was 37.8, 25.4, and 28 %, respectively. The prevalence of high EE, DP, and reduced PA was 20.5, 7.6, and 17.8 %, respectively. The overall prevalence of burnout syndrome among the HIV/AIDS healthcare workers was 76.9 %. Comparing the scores of the three dimensions between the HIV/AIDS healthcare workers and other healthcare workers, the HIV/AIDS healthcare workers’ scores of the dimensions of EE (*F* = 6.350, *p* = 0.012) and DP (*F* = 8.533, *p* = 0.004) were significantly higher than other healthcare workers, but there were no significant differences noted in the dimension of reduced PA (*F* = 0.865, *p* = 0.353; Table 1).

**Table 1:** Comparison of burnout between HIV/AIDS healthcare workers and other healthcare workers

Variables	HIV/AIDS healthcare workers	Other healthcare workers	*F*	*p*
EE	12.11 ± 5.31	10.56 ± 5.00	6.350	0.012
DA	6.75 ± 4.59	5.29 ± 3.85	8.533	0.004
PA	16.51 ± 7.67	15.92 ± 5.99	0.865	0.353

### Comparison of mental health

When compared with other healthcare workers, the HIV/AIDS healthcare workers scored higher in SCL-90 total scores. Chi-square tests showed that the number of positive items were significantly different between HIV/AIDS healthcare workers and other healthcare workers (*χ*^2^ = 4.413, *p* = 0.036). As shown in Table 2, there was a significant difference in comparison of depression (*F* = 4.355, *p* = 0.038) and hostility (*F* = 3.959, *p* = 0.047) between HIV/AIDS healthcare workers and other healthcare workers.

**Table 2:** Comparison of SCL-90 scale between HIV/AIDS healthcare workers and other healthcare workers

Variables	HIV/AIDS healthcare workers	Other healthcare workers	*F*	*p*
SOM	1.58 ± 0.62	1.53 ± 0.56	1.238	0.267
O-C	1.83 ± 0.73	1.71 ± 0.60	3.047	0.082
INT	1.59 ± 0.66	1.52 ± 0.50	1.934	0.165
DEP	1.66 ± 0.67	1.53 ± 0.56	4.355	0.038
ANX	1.54 ± 0.60	1.52 ± 0.55	0.837	0.361
HOS	1.61 ± 0.71	1.48 ± 0.53	3.959	0.047
PHOB	1.33 ± 0.53	1.26 ± 0.43	2.336	0.127
PAR	1.47 ± 0.60	1.39 ± 0.43	2.481	0.116
PSY	1.41 ± 0.53	1.36 ± 0.45	1.630	0.203
SLE	1.63 ± 0.66	1.62 ± 0.63	0.305	0.581

### Prevalence of job satisfaction

Nearly one-fourth (24.6 %) of HIV/AIDS healthcare workers were satisfied with their work, 54.9 % were moderately satisfied, and 20.5 % were dissatisfied.

### Factors associated with burnout

On the basis of a bivariate logistic model, the possibility of having burnout symptoms was significantly higher in HIV/AIDS healthcare workers who were dissatisfied with their job, had serious somatization, interpersonal sensitivity, poor quality of sleep, high scores of psychoticism, and used negative coping styles frequently (Table 3).

**Table 3:** Factors associated with burnout among HIV/AIDS healthcare workers

Scale	B	S.E	P	OR	95 % Cl	
					Lower	Upper
Job satisfaction	1.043	0.318	0.001	2.837	1.521	5.291
Somatization	1.306	0.646	0.043	3.693	1.041	13.100
Interpersonal sensitivity	2.740	0.860	0.001	15.493	2.873	83.547
Sleep	−1.295	0.534	0.015	0.274	0.096	0.780
Psychoticism	0.328	0.116	0.005	1.388	1.106	1.742
Negative coping	0.057	0.028	0.039	1.059	1.003	1.118
Constant	−9.675	2.362	0.003	0.000		

## Discussion

The overall prevalence of burnout syndrome among the HIV/AIDS healthcare workers was 76.9 %. The prevalence of low EE, DP, and reduced PA was 41.7, 67, and 54.2 %, respectively. The prevalence of medium EE, DP, and reduced PA was 37.8, 25.4, and 28 %, respectively. The prevalence of high EE, DP, and reduced PA was 20.5, 7.6, and 17.8 %, respectively. Our study reported that HIV/AIDS healthcare workers exhibited a high level of EE, which is considered the core symptom of burnout. Gabbe et al. [3] reported a similarly high rate (56 %) of EE among obstetricians and gynecologists. Indeed, there were significant differences noted in the dimensions of EE and DP between HIV/AIDS healthcare workers and other healthcare workers. These differences may be explained by suggestions that the risk of contracting HIV among HIV/AIDS healthcare workers causes tension and fear. In addition, AIDS patients need more specialized clinical care, while shortages of healthcare workers leads to long-term work overload.

The present results show that HIV/AIDS healthcare workers scored higher in total scores and positive items when compared with other healthcare workers. Comparing the 10 factors in SCL-90, we found that the scores of depression and hostility significantly differed between HIV/AIDS healthcare workers and other healthcare workers, which is similar to previous report [22]. A previous study showed that some healthcare workers in special departments experience more psychological pressure, and the level of mental health was lower, the somatization, depression, obsessive-compulsive, and hostility scores among physicians and nurses in infectious diseases and emergency departments were significantly higher than other departments [37]. A possible explanation for this result is that the more attention AIDS prevention and treatment receives, the greater the responsibility HIV/AIDS healthcare workers assume. Healthcare workers not only provide some medical services, but also medical care and social support for AIDS patients and their families, leading to a heavy workload, overtime, and complex work content, which could cause high levels of psychological stress among HIV/AIDS healthcare workers [13]. Some studies have reported that healthcare workers experience a higher level of work-related pressure compared with the general population, which is an important fact that affects the mental health of health workers [14, 38]. Davhana [8] reported that 89 % of HIV/AIDS healthcare workers found seeing patients suffer and die were very difficult; such an experience made them feel powerless, as the patients would still die, regardless of the treatment they were given. Furthermore, because of this special occupational context and negative factors, HIV/AIDS healthcare workers are more likely to exhibit a negative reaction mode, and gradually produce some negative psychological manifestations, such as irritability, lack of tolerance to work, anxiety, and depression [13]. It not only has an impact on the quality of HIV service, but also a potential threat to the well-being of HIV/AIDS healthcare workers, such as exacerbating the risk of work-related injuries, including the risk of contracting HIV. Therefore, a series of comprehensive measures should be implemented to improve the level of mental health among HIV/AIDS healthcare workers.

HIV/AIDS healthcare workers are a specific occupational group that often needs to deal with people living with HIV and AIDS patients, have more tedious work, long working hours, and irregular work and life time in contrast with other healthcare workers. Thus, HIV/AIDS healthcare workers are more likely to experience burnout because of their work and stress with such characteristics of professional background. Our study showed that burned-out HIV/AIDS healthcare workers have poor job satisfaction, serious somatization, interpersonal sensitivity, poor quality of sleep, high scores of psychoticism, use of negative coping styles frequently. Job satisfaction describes how people feel about their jobs—whether they like or dislike their jobs [16]. In our study, over one-fifth of HIV/AIDS healthcare workers were dissatisfied with their work. HIV/AIDS healthcare workers who were dissatisfied with their job were more likely to experience burnout. Previous research has demonstrated a negative correlation between job satisfaction and EE and cynicism, and a positive correlation with reduced PA [22]. Moreover, our findings suggest that a poor quality of sleep and somatization, described as physical discomfort, were associated with burnout. Due to the increasing workload and erratic time, the HIV/AIDS healthcare workers have somatic symptoms and a poor quality of sleep, and are more prone to have a negative attitude to their work. In addition, some studies have indicated that individuals with interpersonal sensitivity may have senses of discomfort and inferiority, and lack of proper communication with others when they are in contact with people [18]. The HIV/AIDS healthcare workers with symptoms of interpersonal sensitivity have more stress and feelings of isolation due to discrimination and a lack of understanding from others, experiencing distress at work [39]; thus, they are more likely to experience burnout. In addition, previous study have demonstrated that personality traits have an impact on burnout [21]. In the present study, a high psychoticism score was a risk factor for burnout, which suggests that the personality trait of HIV/AIDS healthcare workers exhibited feelings of isolation, lack of emotional investment, indifference to others, stubbornness, and difficulty adapting to the external environment may be more prone to burnout [38]. This study indicated that negative coping is also a risk factor for burnout among HIV/AIDS healthcare workers. This result is similar with the results of a previous study [14]. Günüsen et al. [40] found that burnout levels of nurses could be reduced by means of strengthened psychological and sociocultural dimensions through coping groups.

In summary, occupational burnout and the mental health of HIV/AIDS healthcare workers should be taken seriously by authorities, in particular, the high AIDS epidemic province, such as Guangxi. Relevant departments should try to meet the reasonable material and spiritual needs of HIV/AIDS healthcare workers, improve welfare to stimulate their enthusiasm for work, and reduce the occurrence of burnout. Furthermore, enhancing education on reducing HIV-related stigma and discrimination, actively seeking countermeasures, and improving the level of mental health to alleviate psychological pressure is warranted. Moreover, departments should raise awareness of occupational protection and effective protective measures should be taken to reduce the occurrence of occupational exposure and improve workplace safety. The above measures can improve work efficiency of HIV/AIDS healthcare workers and enhance the prevention and treatment of AIDS.

### Limitations

Despite these merits, there were several limitations in the present study. The first limitation is the potential for sampling bias, as the subjects in the present study were recruited from four hospitals that treated HIV/AIDS in Guangxi province, which might not be representative of HIV/AIDS healthcare workers throughout China. The second limitation involves the weak causal inference because this study had a cross-sectional design. Therefore, future studies should include a longitudinal follow-up to determine the relationships between burnout and other factors.

## Conclusions

Burnout is highly prevalent in HIV/AIDS healthcare workers, with 76.9 % of HIV/AIDS healthcare workers meeting the accepted criteria for burnout. Moreover, the HIV/AIDS healthcare workers’ scores of the dimensions of EE and DP were significantly higher than other healthcare workers, These findings indicate that some effective interventions for HIV/AIDS healthcare workers should be designed to address burnout. In addition, HIV/AIDS healthcare workers experience lower levels of psychological health, compared with other healthcare workers. In the present study, low job satisfaction, serious somatization, interpersonal sensitivity, poor quality of sleep, high psychoticism scores, and using a negative coping style frequently were associated with high burnout. Interventions should be targeted at reducing the occurrence of burnout and alleviating psychological pressure.
